# Clinical Decision Support Framework for Segmentation and Classification of Brain Tumor MRIs Using a U-Net and DCNN Cascaded Learning Algorithm

**DOI:** 10.3390/healthcare10122340

**Published:** 2022-11-22

**Authors:** Nagwan Abdel Samee, Tahir Ahmad, Noha F. Mahmoud, Ghada Atteia, Hanaa A. Abdallah, Atif Rizwan

**Affiliations:** 1 Department of Information Technology, College of Computer and Information Sciences, Princess Nourah bint Abdulrahman University, P.O. Box 84428, Riyadh 11671, Saudi Arabia; 2 Department of Computer Science, COMSATS University Islamabad, Attock Campus, Attock 43600, Pakistan; 3 Rehabilitation Sciences Department, Health and Rehabilitation Sciences College, Princess Nourah bint Abdulrahman University, P.O. Box 84428, Riyadh 11671, Saudi Arabia; 4 Department of Computer Engineering, Jeju National University, Jejusi 63243, Republic of Korea

**Keywords:** clinical decision, U-Net, CNN, CAD system, brain tumor, classification, segmentation

## Abstract

Brain tumors (BTs) are an uncommon but fatal kind of cancer. Therefore, the development of computer-aided diagnosis (CAD) systems for classifying brain tumors in magnetic resonance imaging (MRI) has been the subject of many research papers so far. However, research in this sector is still in its early stage. The ultimate goal of this research is to develop a lightweight effective implementation of the U-Net deep network for use in performing exact real-time segmentation. Moreover, a simplified deep convolutional neural network (DCNN) architecture for the BT classification is presented for automatic feature extraction and classification of the segmented regions of interest (ROIs). Five convolutional layers, rectified linear unit, normalization, and max-pooling layers make up the DCNN’s proposed simplified architecture. The introduced method was verified on multimodal brain tumor segmentation (BRATS 2015) datasets. Our experimental results on BRATS 2015 acquired Dice similarity coefficient (DSC) scores, sensitivity, and classification accuracy of 88.8%, 89.4%, and 88.6% for high-grade gliomas. When it comes to segmenting BRATS 2015 BT images, the performance of our proposed CAD framework is on par with existing state-of-the-art methods. However, the accuracy achieved in this study for the classification of BT images has improved upon the accuracy reported in prior studies. Image classification accuracy for BRATS 2015 BT has been improved from 88% to 88.6%.

## 1. Introduction

Multiple stakeholders are involved in providing intelligent healthcare, including medical professionals, patients, hospitals, and academic research institutions. It is an organic totality that incorporates numerous dimensions, such as the prevention and monitoring of diseases, the diagnosis and treatment of illnesses, the management of hospitals, the making of decisions regarding health care, and the conducting of medical research [1]. Smart healthcare employs technology such as wearable sensors, artificial intelligence, and the internet to dynamically access healthcare-related information. This information is then used to intelligently control and respond to medical ecosystem demands. Smart healthcare can encourage interaction among all healthcare stakeholders, ensuring that participants receive the services they require, assisting parties in making informed decisions, and facilitating the logical allocation of resources [2]. Smart healthcare’s utilization of these technologies has the potential to drastically lower healthcare costs and risks while simultaneously making tailored medical treatment the norm [3]. Clinical decision support systems, disease prevention and risk monitoring, and patient self-management [4] are some examples of the services that may be provided by smart healthcare systems. Artificial intelligence (AI) has the ability to improve the quality and safety of clinical decision-making systems. Currently, AI is successfully integrated into decision support systems for diagnosis in data-intensive disciplines, such as pathology and radiology. These systems, which include computer-aided diagnosis systems for classifying lesions in a variety of diseases [5,6], are examples of the types of clinical applications that have recently benefited from the application of artificial intelligence.

Tumors of the central nervous system (CNS) are a form of cancer that are uncommon but deadly. These tumors frequently rob patients of their basic quality of life. Patients who have CNS tumors have a poor prognosis, notwithstanding the progress that has been made in our understanding of the disease processes that are associated with CNS tumors. In order to aid in the detection and treatment of CNS malignancies, improved techniques for characterization, diagnostics, and monitoring are required. Utilizing more recent imaging methods is an essential component of neuro-oncology’s toolbox of available options for treatment. Magnetic resonance imaging and computed tomography (CT) are the two imaging techniques that are currently considered to be the best models for the radiographic evaluation of neuro-oncological diseases [7]. Imaging and computer-aided diagnosis systems in medicine assist in avoiding the need for diagnostic biopsies.

The early diagnosis of brain tumors not only saves lives but also reduces the likelihood of disability. With early detection, there will be less manipulation and surgical removal of tissue from the brain, the most delicate organ in the body [8]. The manual diagnosis of the condition requires a radiologist to record a 3D image for initial understanding. Then, a specialist is consulted for image analysis and treatment planning. As reported by Johnson et al. [9], examining the accuracy of manual brain tumor diagnosis indicates that expert reviewers differ. According to reports, between 90% and 95% of professionals agree on the diagnosis of a brain tumor manually. For multiclass classification, such as medulloblastoma, and glioma, the experts’ agreement drops to 77% and 58%, respectively [9]. As a result, developing AI-based assistive tools for accurate detection of brain tumors is required.

MRI is recommended for the CAD system of brain tumors, because there is no risk of ionizing radiation and it can identify blood flow in veins correctly [10]. Several strategies for CAD systems for brain tumors have been developed in recent years, including conventional machine learning (ML) [11] and deep learning (DL) [12,13,14]. The identification, segmentation, and classification of brain tumors have been the focus of numerous research efforts to date, but studies in this field are only getting started. The comprehensive review conducted by Ali et al. [15] of the relevant literature revealed that deep learning technology ultimately produces fantastically realistic performances in the processing of brain tumor images. Classical machine learning, such as the support vector machine (SVM) is a dominant method for the classification of brain MRI. Deep learning algorithms, on the other hand, are the most successful, with deep convolutional neural networks leading the pack.

The primary modules of the CAD system for detecting brain cancers are image preprocessing and enhancement, brain skull stripping, brain tumor segmentation, feature extraction and selection, and classification of benign and malignant tumors [16,17,18]. The most crucial stage in the entire CAD system is brain image segmentation, which affects the yielded accuracy [19]. Image segmentation is the process of extracting regions of interest (ROIs) from 3D image data (MRI). The primary purpose of segmenting these data is to identify parts of the anatomy that are needed for a specific study [16]. Manual segmentation for a huge number of MRIs is time-consuming, but new advancements in AI techniques are making it quicker to execute regular tasks [20,21]. This study introduces deep learning-based automatic segmentation of brain MRI images for developing a computer-aided diagnosis of brain tumors—gliomas. Specifically, we propose a modified version of the U-Net convolutional neural network architecture that should improve the performance of automatic brain MRI segmentation. The architecture of U-Net convolutional neural networks has been used in this study since it has demonstrated superior performance in comparison to the present state of the art in the segmentation of MRI images [22]. The phase of image segmentation is preceded by automatic feature extraction and classification, both of which are accomplished through the utilization of a five-layer convolutional neural network.

This article proposes a lightweight, effective U-Net deep network implementation, with the ultimate goal being precise real-time segmentation. To do this, U-Net’s input layer had to be modified to accommodate images of a smaller resolution. In contrast to the initial version of U-Net, where input images were always 572 by 572 pixels in size, we have used images as small as 32 × 32 pixels. The U-Net model accepts images of 32 × 32 pixels. The encoding part of the U-Net model decreases the pixels of the image and extracts tiny features from low-resolution images. The U-Net model, which consists of an encoder and a decoder, is utilized for the segmentation stage, while the CNN model is employed for the classification. We believe that our method is more efficient and less time-consuming than competing methods. Our approach was tested and proven accurate on a benchmarking dataset—BRATS 2015.

In order to highlight the significance of the work that we are introducing, the following is a list of the contributions provided by the present study:

- Introducing an enhanced lightweight and effective U-Net deep network architecture, with the ultimate goal being precise real-time segmentation of brain MRIs.
- A simplified CNN-based architecture for BT classification is presented for automatic feature extraction and classification of the extracted regions of interest.

The manuscript is organized as follows. Section 2 is devoted to the literature review. In Section 3, the suggested methodology, proposed framework, and description of the data used are provided. The findings are analyzed in Section 4. Section 5 and Section 6 present the study’s conclusion and suggestions for future research, respectively.

## 2. Literature Review

Healthcare organizations are starting to implement AI and related technologies, since they are becoming increasingly common in industry [23,24,25,26,27] and society broadly [28]. Many areas of patient care and administrative procedures within provider, payer, and pharmaceutical organizations stand to benefit from these innovations [29]. Medical diagnosis [30,31,32] is an area where numerous studies have shown AI to be on par with or even superior to human practitioners. ML/DL algorithms [33,34,35] are currently more accurate than radiologists in detecting cancerous tumors, and they are also helping researchers figure out how to build study populations for expensive clinical trials. Machine learning is a branch of AI that is dedicated to constructing methods that “learn from data to improve performance on some set of tasks.” Machine learning is one of the most widespread types of AI: 63% of companies surveyed in 2018 [36] whose organizations were already pursuing AI used machine learning in their operations [26,29]. The most prevalent use of conventional machine learning in the healthcare field is precision medicine, which predicts the treatment protocols that are likely to be effective on a patient based on numerous patient traits and the treatment context [37]. The vast number of machine learning and precision medicine solutions need a training set with predefined outcome variables—supervised learning. The foundation of machine learning and deep learning in medical imaging is the artificial neural network (ANN). Layers of nodes in an ANN can range from the hundreds to the millions. Deep learning makes use of multilayered artificial neural networks (for example, >8), and is generally considered a more advanced version of ML that can analyze more data and sophisticated inputs [38]. Each node is fed by data from the other nodes, and the contributions of each node are taken into account when calculating the final score. When used properly, the ANN should increase the proportion of right responses.

Because of the increased computing power of today’s graphics processing units (GPUs), such models may contain thousands or even tens of thousands of features that have been concealed from view. The detection of possibly malignant tumors in medical imagery is one of the most common applications of deep learning in the healthcare industry [6,39,40,41,42,43,44,45]. Radiomics, the detection of clinically significant patterns in imaging data that are invisible to the naked eye, is becoming an increasingly popular use of deep learning [46]. Oncology-specific image analysis typically employs both radiomics and deep learning. Together, they offer improved diagnostic precision and accuracy in CAD systems.

Many researchers have lately advocated the use of artificial intelligence to automatically discover and diagnose brain abnormalities in MRI scans. Table 1 summarizes the present state of the field and the weaknesses of each strategy based on recent research that has used ML/DL algorithms to detect brain cancers. The table helps to highlight the most important features of the new system. The table helps to highlight the most important features of the new system.

Amulya and Prathibha [47] proposed an ML-based method for distinguishing between tumors and non-tumors in brain MRI images by making use of a KNN machine learning algorithm and extracting features using scale invariant feature transform (SIFT) and speeded up robust features (SURF). They conducted their experiments on a total of 101 MRI scans obtained from the MR-TIP database. Accuracy was 94% when utilizing SURF alone and 96% when using SURF and SIFT together. They conducted experiments using several classifiers, including the fuzzy C-mean and SVM, but found that their own proposed technique yielded the best results overall. The detection of brain tumors using another classical ML-based method was introduced by Virupakshappa and Amarapur [48]. Gabor wavelets [49] and a statistical feature extraction method were used to extract the texture features. When separating objects, they turned to the fuzzy C-mean method. One hundred MRIs were utilized to evaluate the system, with sixty images used for testing and forty used for training. Artificial neural networks (ANNs) were used for tumor classification. The performance of the system was measured using precision, recall, and accuracy, with an overall accuracy of 85% being achieved in this study.

Raj et al. [50] have created a system for the classification of MRI obtained from District Hospital Palakkad, Department of Neurosurgery, India. They have applied four phases on the images: image preprocessing, segmentation, feature extraction and classification of brain tumors. For segmentation, they used K-means clustering. For feature extraction, the GLCM and Gabor filters were used, and KNN was used to classify brain tumors. The accuracy was determined by comparing the obtained results to ground truth values, and this study achieved 95% accuracy. Ahmet et al. used novel techniques for brain tumor detection [51]. The primary goal of this study was to clearly identify the tissue that had been damaged by cancer. For preprocessing, they used morphological operations. The threshold-based approach was used for segmentation, and the median filter was used for filtering. In this study, 100 MR images from the TCIA database were used. They achieved a 96% average accuracy. Devkota et al. [52] proposed a CAD method for early-stage detection of brain tumors. They segmented 19 brain MR images affected by four different types of tumors (glioma, metastatic adenocarcinoma, meningioma and sarcoma). The median filter was used for preprocessing, and Mathematical morphological operations were used for MR image segmentation. They classified brain tumors using SVM and achieved an accuracy of 92% on a small dataset.

The work done in [53] presented a framework for brain tumor classification in MRIs. For feature selection, they combined 2D DWT and 2D Gabor Filter techniques. Backpropagation neural network classifier was used to classify such tumors as meningioma, glioma, and pituitary. They used 3064 slices of T1-weighted MRI scans. They achieved an overall accuracy of 91.9% for meningioma, glioma, and pituitary. In [54], a novel method for brain tumor classification and segmentation using genetic algorithms was introduced. For preprocessing, adaptive constraint enhancement was used, and for enhancement, skull scripting was used. A total of 22 images from the DICOM dataset and 44 images from the web brain dataset were used in the analysis. They used various techniques in this paper, including watershed segmentation, FCM, direction cosine transform (DCT) segmentation, and bakery wavelet transform (BWT), and the technique with the highest segment score was chosen. They were 92.03% accurate.

Rajesh et al. proposed a new method for feature extraction based on rough set theory in [55]. For preprocessing, differential-based adaptive filtering (DAF) was used, and the region growing algorithm was used for segmentation. For classification, the PSONN technique was used. The MRI dataset comprised 90 MR images, 60 of which were tumorous and 30 of which were not. The data were obtained from the Government Medical College Hospital in Trivandrum, India. Sensitivity, specificity, and accuracy were used as evaluation metrics to assess the system’s performance. They achieved a 96% accuracy rate. In [56], Shree et al. proposed a method for detecting brain tumors. PNN was used to classify tumors from MR images. In this study, two datasets were used: one for training and one for testing. The training dataset was obtained from the DIACOM website. They removed noise and smoothened the images during preprocessing. DWT was used for image decomposition, and GLCM was used to extract textural features. On the test dataset, 95% accuracy was achieved.

Recent research has shown that the CAD system for brain tumors can be much improved with the help of deep learning algorithms [57,58,59,60,61,62,63,64,65]. Brain images were classified into four types of tumors using a DCNN [66]. Fuzzy C-means was used to segment the images, and DWT was used to extract the features. PCA was used to reduce the features. The sevenfold cross-validation technique was used for classification and training of the seven-hidden-layer DNN model. The dataset used in this study comprised 66 brain MRIs with four types of brain tumors: normal, sarcoma, metastatic bronchogenic carcinoma, and glioblastoma. It was obtained from the website of Harvard Medical School. Recall, precision, F-measure, classification rate, and area under the curve were used to evaluate the proposed method’s performance. They achieved an average classification rate of 97.96% for all four tumor classes. A CNN-based deep learning algorithm was used to classify tumor type from a collection of MRIs [67]. In this study, three different datasets were used: REMBRANDT from Cancer Imaging, Brain Images of Normal Subjects (BRAINS) Image Bank repository of the University of Edinburgh, and MIRIAD. The images are classified into five categories: astrocytoma, glioblastoma, oligodendroglioma, healthy tissue, and unknown tumor. The overall average F1 score was 99.46%. Jude et al. introduced a modified architecture of the DCNN model [68] for the classification of abnormal brain MRI scans. They used an assignment method for estimation of weights in the fully connected layer instead of gradient descent to update the weights, and modification in the conventional DCNN was performed in this layer. They used 220 brain MR scans that were obtained from M/s. Devaki Scan Centre in order to carry out the experiment, and as a result, they were able to achieve an accuracy rate of 96.4%.

We have compared the proposed U-Net–CNN approach to other studies that have used the same dataset, BRATS 2015, to better illustrate the unique features of our proposed end-to-end segmentation and classification system for brain tumor MRIs. With this, we were able to gauge the significance of the U-Net–CNN method. BRATS 2015 is a challenging dataset encompassing many types of brain MR images. The BRATS challenge was held in connection with an international conference on medical image computing (MICCAI) to assess the present state of the art in automatic BT segmentation and to compare alternative methodologies. The BRATS dataset was generated for this aim as a one-of-a-kind collection of MR scans of LGG and HGG glioma patients with successive manual tumor categorizations by different human experts. Quantitative assessments demonstrated significant disagreement among human raters in segmenting distinct cancer subregions (Dice ratings ranging from 74% to 85%), highlighting the difficulties of this task. As a result, much effort has been expended on this benchmarking dataset. Some of them are mentioned in Table 1 and reviewed in this section to demonstrate the current state of the art for the accuracies attained with it to the present time. In 2015, Vinay Rao [69] suggested a DCNN model for brain tumor classification and segmentation. For this investigation, they employed the BRATS 2015 dataset. They used pixel-by-pixel categorization. Each pixel joins to generate a multimodal image based on its surroundings. They employed a stochastic gradient to classify each pixel that was surrounded by patches. ReLu was employed in association with the final hidden layer to enhance gradients and obtained 67% accuracy.

In 2016, Pereira et al. [70] used the DCNN for tumor segmentation and detection using BRATS 2015 data. They acquired a greater sensitivity rate of 86% by using neural network hyperparameters such as dropout, leaky rectifier linear units, and tiny convolutional kernels. They trained two architectures, one for each, to ensure the HGG and LGG. After combining LGG and HGG, they reached 0.87 DSC for the entire tumor (LGG, and HGG together). Utilizing data from the BRATS 2015 challenge, Casamitjana et al. [71] developed a 3D convolutional neural network (CNN) for segmenting BT. This work established a robust connection between three separate models. Two fully convolutional 3D CNN architectures were presented, both of which took their inspiration from popular 2D models employed for generic image segmentation. A third model was trained, and it was a two-pathway deep medic network variation. By subtracting the volume’s mean and dividing by the volume’s standard deviation, they normalized the data inside each input volume. Training data with up-sampling layers can enhance effective batch size with low memory and computational costs. They achieved 84% for the Dice score for segmenting the whole tumor subregion of single-resolution images.

In 2017, Dong et al. [72] introduced the U-Net framework for the segmentation of the BRATS brain MR images dataset. This study made use of the BRATS 2015 dataset, which included HGG and LLG. They employed fivefold cross-validation and obtained a DSC of 0.86 for the combined results of HGG and LGG to properly identify the entire tumor. Mengqiao et al. suggested a 3D CNN for glioma segmentation [73]. They developed a 22-layer network and employed leaky rectifier linear units as activation functions in each activation layer until the last one, which used softmax as an activation function. The model was trained using 20 MR pictures and tested using 100 MR images collected from the BRATS challenge repository. DSC, PPV, and sensitivity were utilized to evaluate the model, and they reached 0.84, 0.88, and 0.82 for the entire tumor region, respectively.

In 2018, Wang et al. [74] employed CNN to detect brain tumors with an accuracy of 80%. This investigation was carried out using a dataset of 480 MR images obtained from the BRATS challenge. A total of 320 MR scans were used for training and 160 for testing. They preprocessed the input before submitting them to the CNN. They eliminated noise and tissue that were not part of the brain during preprocessing and then improved the contrast to assure the quality of medical images. They employed active contour to separate the tumor area from the surrounding areas, and the segmented images were then fed into CNN for classification. In this study, fourfold cross-validation was performed and the attained sensitivity, specificity, and accuracy were 85.7%, 86.5%, and 86.0%, respectively. In [75], Cui et al. introduced an automatic segmentation method from MRI data containing brain gliomas. The work that was proposed was founded on a cascaded deep learning convolutional neural network. This network was comprised of two subnetworks: a tumor localization network (TLN) and an intratumor classification network (ITCN). Determining the location of the tumor on an MRI slice was the primary objective of the first subnetwork. After that, the ITCN was utilized to assign labels to multiple subregions within the previously defined tumor region. On the datasets from BRATS 2015, the proposed method was evaluated. On the combined HGG and LGG MRIs, the DSC and sensitivity values obtained from the experiments came in at 0.89 and 0.80, respectively. A CNN-based classification model for brain tumors was introduced through the work of Lang et al. [76]. This method combines the CNN model that was optimized with the SVM model in order to make full use of the strengths of both methods. The newly proposed method was tested on the BRATS 2015 database, and the results showed that it had an accuracy of 0.88%. Li et al. [77] developed a U-Net-based model for the segmentation of MRIs taken of brain tumors. The newly presented model is capable of automatically generating segmentation maps slice by slice. The proposed model has been shown to be accurate by both the BRATS 2015 and BRATS 2016 studies. The experimental results have achieved a DSC of 0.89 for the BRATS 2015 training dataset and 0.87 for the BRATS 2017 training dataset.

In 2019, Peng et al. developed a deep learning model for segmenting brain tumor MR images. The model is built on a 3D U-Net architecture that employs many U-Net blocks to record long-distance spatial features at various resolutions. They attained Dice scores of 0.85, 0.72, and 0.61 for the whole tumor, tumor core, and enhancing tumor, respectively, on the BRATS 2015 testing set.

**Table 1:** A summary of the current state of the art and the limitations of each technique.

Author	Techniques	Dataset/Database	Strengths	Weaknesses
Amulya and Prathibha [47]	SURF, SIFT, KNN	101 Brain MRIs, MR-TIP and overcode.yak.net	In order to perform well, deep networks need very huge datasets. Classical ML methods typically perform better than deep networks on smaller datasets. Classical ML algorithms are straightforward to comprehend and interpret because to their reliance on direct feature engineering. In addition, with a deeper comprehension of the data and algorithms, tweaking hyper-parameters and making other changes to the model designs is much less of a challenge. However, deep networks have a lot of “black box” characteristics, meaning that their “insides” are not fully understood even now by academics. Because of this absence of theory, dealing with hyper-parameters and designing networks is also difficult.	The significant features are extracted from the input image using some feature extraction algorithm, and the classical machine learning model is trained to recognize the tumor from normal tissues. This is just one of the three steps that make up the framework for classifying brain tissues into normal/abnormal ones. Algorithms for extracting features, edge-related characteristics, and other required information might be time-consuming to run. This is especially true when the lines between healthy tissue and tumors are unclear or fuzzy, as is the case with many cancers.
Virupakshappa and Amarapur [48]	FCM, Gabor Wavelet, ANN	60 MR Images Source was not mentioned		
Raj et al. [50]	K-mean, GLCM Gabor Filter, KNN	T1-weighted MRIs, District Hospital Palakkad, Department of Neurosurgery, India		
Ahmet [51]	Morphological Operations, Threshold-based Segmentation, Median Filter	100 MR Images, TCIA		
Shree et al. [56]	PNN, DWT, GLCM	DICOM		
Devkota et al. [52]	Median filter, Mathematical Morphological Operations, SVM	19 MR Images, Source was not mentioned		
Ismael et al. [53]	DWT, Gabor Filter, BPNN	3064 T1-weighted MRI scans, Figshare		
Bahadure et al. [54]	Watershed Segmentation, FCM, Direction Cosine Transform Segmentation, Bakery Wavelet Transform, Genetic Algorithm	22 MRIs from DICOM, 44 MRIs from Web Brain		
Rajesh et al. [55]	Differential based Adaptive Filtering (DAF), Region Growing Algorithm, PSONN	90 MRI scans, Government Medical College Hospital, Trivandrum, India		
Mohsen et al. [66]	DCNN, DWT, PCA, FCM	66 MRIs, Harvard Medical School Website	There is no requirement for feature engineering with DL-based methods. In contrast to traditional ML algorithms, deep learning methods can be readily customized to serve a variety of purposes and domains.	The following deficiencies are common to most DL-based methods: 1. Firstly, the classification systems based on convolutional neural networks (CNNs) have a complex structure that necessitates a lot of computing power. 2. The present state of the art for segmentation and classification on the aforementioned BRATS 2015 dataset demonstrates poor to medium segmentation and classification’s performance.
Balasooriya et al. [67]	CNN	REMBRANDT BRAINS MIRIAD		
Hemanth et al. [68]	Modified DCNN	220 Brain MRIs, M/s. Devaki Scan Centre		
Vinay Rao et al. [69]	CNN	BRATS 2015		
Sergio Pereira et al. [70]	DCNN	BRATS 2015		
Casamitjana et al. [71]	3D CNN	BRATS 2015		
Dong et al. [72]	U-Net	BRATS 2015		
Mengqiao et al. [73]	3D CNN	BRATS 2015		
Heng wang [74]	CNN	BRATS 2015		
Cui et al. [75]	Cascade DCNN	BRATS 2015		
Lang et al. [76]	CNN, SVM	BRATS 2015		
Li et al. [77]	Modified U-Net model	BRATS 2015		
Peng et al. [78]	3D U-Net model	BRATS 2015		
Proposed method	U-Net and CNN Cascaded framework	BRATS 2015	In this work, we present a lightweight and easy-to-use end-to-end segmentation and classification system for BT MRIs. Precision in real-time segmentation was the driving force behind the introduction of a lightweight, effective implementation of the U-Net deep network. Further, we provide a simplified CNN-based architecture for BT classification in an effort to enhance performance.	

## 3. Materials and Methods

### 3.1. Clinical Brain Image Dataset

The data used in this work were obtained from a public dataset—the Multimodal Brain Tumor Segmentation Benchmark (BRATS 2015) [79]. The clinical image data are comprised of 65 multicontrast MR scans obtained from glioma patients [80]. Magnetic resonance imaging (MRI) is one of the most commonly utilized medical imaging analysis techniques for diagnosing problems of the nervous system [81] and the brain due to its outstanding soft tissue resolution and lack of potentially dangerous radiation effects [82]. Since each glioma is unique in terms of its size, form, and structure, several MRI sequences, such as T1-weighted, T1c, and T2-weighted, as well as FLAIR, are utilized in order to examine the tumors. These sequences are analyzed to determine the various subregions of the tumor and offer information relating to those findings. Therefore, appropriately segmenting gliomas and the structures found inside the tumor through the application of various MRI sequences is important for research and can provide extra assistance to medical professionals when formulating a diagnosis strategy.

A total of 65 multicontrast MR scans from glioma patients make up the clinical image data utilized in this study, 14 of which were taken from patients with low-grade gliomas (astrocytomas or oligoastrocytomas) and 51 from patients with high-grade gliomas (anaplastic astrocytomas and glioblastoma multiforme tumors). The scans were taken both before and after treatment, with resections visible in two of the volumes. They were gathered over the course of several years from four institutions (Bern University, Debrecen University, Heidelberg University, and Massachusetts General Hospital) using MR scanners from four different vendors, with two different field strengths of 1.5T and 3T, and two different implementations of the imaging sequences—2D and 3D.

The training dataset includes the ground truth as an input. The image dataset is found in the file format known as Meta Image (.mha). All of the images have already had the skull removed, and the resolution in all of the MRI sequences is very clear. Within this particular dataset, each patient undergoes a total of four MRI sequences. The acronyms FLAIR, T1-weighted, T1c, and T2-weighted describe these images. Necrosis, edema, enhancing, and nonenhancing are the four categories that are used to classify the tumor tissue in this set of data. Image processing techniques can be used to identify the different class regions, since each region in the class has unique radiological features that can be distinguished from one another. Different sequences can provide crucial information about the different intratumoral locations due to the complicated nature of gliomas. The quantitative evaluations were carried out on the basis of three different tumor regions: the whole tumor, the core tumor, and the enhancing tumor. Complete tumor consists of all tumor regions, including necrosis, edema, enhancing, and nonenhancing, while core tumor consists of all three regions save edema, and enhancing tumor consists of only enhancing region. The BRATS 2015 dataset includes a total of 220 MRIs of high-grade gliomas as well as 54 MRIs of low-grade gliomas. FLAIR, T1, T1-contrast, and T2 are the four different MRI sequences that can be performed on each individual participant. For the purpose of determining how well our model works, we employ a 10-fold cross validation. Specifically, the data are divided at random into three sets: training, validation, and testing, with 70%, 15%, and 15% the percentages of division between the training, validation, and testing sets, respectively. In Table 2, we list the total number of images that were used across all three phases of the experiment: training, validation, and testing.

### 3.2. Experimental Setup

Python 3.7.3 (64-bit) is used as the implementation language for this study. Conv3D, MaxPooling3D, UpSampling3D, concatenation, flatten, and dense are some of the Keras layers utilized by the U-Net and CNN model. We used a 16 GB Intel Core i5 machine with a 2.00 GHz x64-based processor.

### 3.3. Proposed Framework

**Figure 1:**
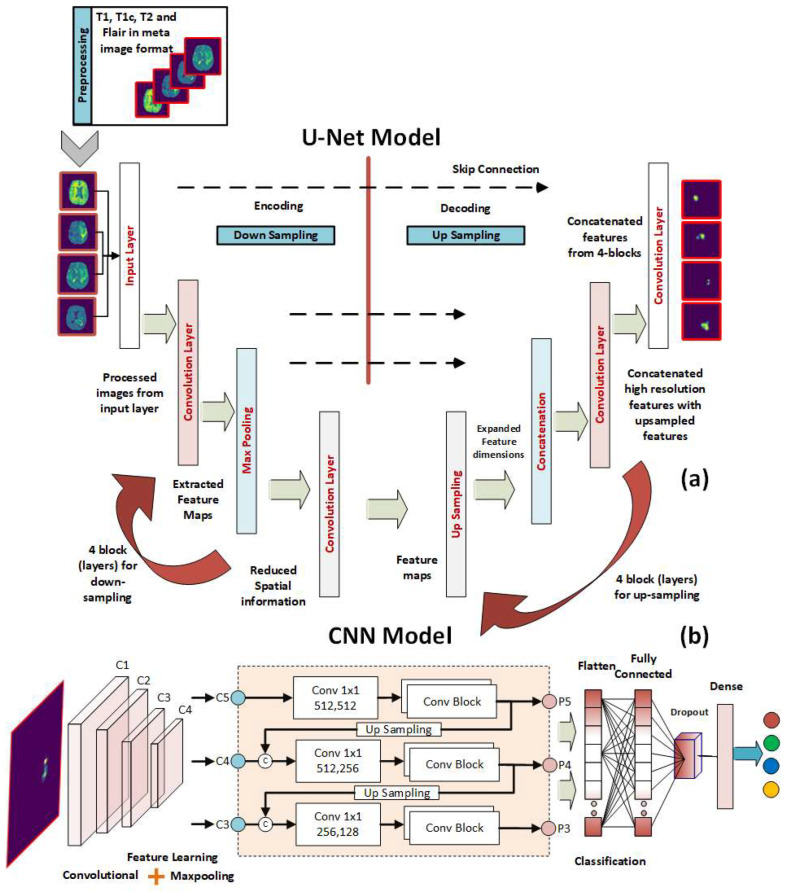
The proposed U-Net and CNN cascaded framework for the segmentation and classification of brain tumor MRIs. (**a**) The U-Net layers for the segmentation of input images. (**b**) The CNN layers for the classification of the segmented ROIs.

In this study, an integrated framework based on U-Net CNN and DCNNs is introduced for CAD of brain MRI images. Such a CAD system involves five main stages including image preprocessing and enhancement, brain skull stripping, brain tumor segmentation, feature extraction and selection, and classification of benign and malignant tumors. The proposed U-Net and CNN cascaded framework for the segmentation and classification of brain tumor MRIs is depicted in Figure 1. During the image preprocessing step, many operations on the data are performed. Some examples of these operations include image scaling, removing noise from an image, and enhancing contrast. Improving the data so that it can be processed further is the goal of this step. The original image resolution for the BRATS data used in this investigation was 240 × 240 × 155. The data contained four different MR sequences: T1, T1c, T2, and FLAIR. After being scaled down to a format measuring 32 × 32 × 32, these images are then provided as input to the segmentation model. The MRI sequences that make up this data are presented as a 3D color image and in the format of Meta Image. The U-Net model is used to automatically segment the preprocessed brain MRIs and hence extract the regions of interest (ROIs). After the ROIs have been automatically extracted, they are used to train and test a deep CNN that can distinguish between tumor and healthy tissue samples.

#### 3.3.1. U-Net Model for Brain MRI Segmentation

The goal of brain tumor segmentation is to automatically and precisely locate a brain tumor in MR images. In contrast to ML [83] and non-AI [84] methods, the DL segmentation methods MRNet [85], and U-Net [86] are fully automated. Further, DL techniques are superior to ML and non-AI approaches when it comes to segment medical images [87,88].

U-Net is a convolutional neural network (CNN) that is fully connected and is utilized for effective semantic segmentation of images [86]. The architecture of U-Net is founded on an autoencoder architecture, which means that the network will duplicate its inputs to its outputs. An autoencoder is a deep NN that compresses the input matrix into a latent-space representation. This is just a demonstration of the images in compact form that indicates which pixels are the closest together. In order to produce an output, the compressed data must first be reconstructed. An encoder and a decoder make up each of the two pathways that make up an autoencoder network. The data is first compressed into a latent-space representation by the encoder, and then the decoder is employed to recreate the inputs from the latent-space representation. U-Net makes use of a convolutional autoencoder architecture, which means that the convolutional layers are utilized both during the encoding process as well as during the decoding process. The input image to U-Net is captured by its encoder route, which contains a stack of convolutional and pooling layers. Accurate localization is achieved by transposed convolutions in the decoder path. U-Net consists solely of stacks of convolutional layers and max-pooling layers—it lacks a fully linked feedforward layer. U-Net can be simply adjusted to function with any image dimension [89], despite being created for 572 × 572 images. Adding many stacked convolutional layers allows the network to acquire more accurate information from the input images with less compression [90].

In this work, an efficient lightweight implementation of U-Net deep networks is proposed with the goal of providing accurate real-time segmentation. This has been accomplished by altering the input layer of U-Net to accept lower sizes of input images. The results have been quite promising. Instead of working with the default size of input images in the original version of U-Net, which was 572 by 572 pixels, we have utilized images of smaller sizes, including 32 by 32 pixels. In addition, we have incorporated a more extensive and deeper stack of convolutional layers into the proposed architecture (four convolutional blocks, where each block has two convolutional layers). This has assisted us in obtaining more precise information from the input images while also reducing the amount of compression.

The architecture of the proposed U-Net model is shown in Figure 1a. Along the encoding path, which is sometimes referred to as the down-sampling path, there is a total of four convolutional blocks. Each block has two convolutional layers, each of which has a filter size of 3 × 3, and they are separated from one another by a stride size of 1. Although the ReLU is typically used as an activation function in convolutional layers, the ELU has been used in this study as an activation function because it is more suitable for use in applications involving image segmentation. This is in contrast to the general practice, which is to use the ReLU. Because of this, the ELU activation function plays a significant role in the segmentation model that we provide. A convolutional layer that contains an activation function has the potential to increase the number of feature mappings from 4 to 64. Every single convolutional block uses a max-pooling layer that has a stride of 2 × 2 in order to cut down on the overall size of the feature maps that are needed for the process. After decoding, a feature map with 32 × 32 pixels is shrunk to one with only 2 × 2 pixels. In addition, the extraction technique makes use of four convolutional blocks to handle the data. At the start of each block, a Conv3D layer is added, and its stride size is set to 2 × 2, and its filter size is set to 3 × 3. The feature maps included in this section range in size from 2 × 2 grids to 32 × 32 grids (the original size of the input image). The feature maps that were generated by the decoding path and the upsampling path are concatenated in a concatenation that occurs later in each block after the convolutional layer has been processed. Padding of a similar nature is utilized in order to keep the spatial dimensions of the input and output layers consistent with one another. Finally, a Conv3d 1 × 1 layer is utilized to partition the original feature map into the tumor region and the rest of the brain. At the final convolutional stage, Sigmoid is employed as the activation function. We tested the network’s functionality at 2, 3, and 4 depths.

#### 3.3.2. DCNN Classification Model

After the ROIs have been automatically extracted using the U-Net model described in the preceding subsection, the ROIs are utilized to train and test a deep CNN that is able to differentiate between samples of healthy and malignant tissue. The deep convolutional neural network has quickly become the most popular tool for usage in the field of image processing due to its better pattern-recognition capabilities [91,92,93,94,95]. CNNs have a variety of different layers. Convolutional layers, pooling layers, and completely connected layers are the most common [96]. The primary layer of a CNN architecture is the convolutional layer. Image features such as edges and colors can be extracted with its help. The dimension of the collected features is reduced through the use of the pooling layer, which reduces the complexity and the processing time. The fully connected layer is the final stage of the CNN model, which aims towards linearity within the networks.

A schematic of the proposed CNN model architecture for BT tissue classification is shown in Figure 1b. The input layer of the proposed model takes in the input images, ROIs, which have the size of 32 × 32. The input layer is followed by a set of convolutional layers. The proposed model contains five convolutional layers to extract the significant features from the ROIs. As segmented images are input to our model, we chose max pooling because we only cared about the most informative features in the tumor tissue. Using a max-pooling layer after each convolutional layer is used to improve the accuracy of the final result. The convolutional layer we utilized has 3 kernels and 2 strides. ReLU is used in all convolution layers. Edges can be protected from data loss by using padding. The padding applied to inputs and outputs is identical. After each convolution layer, a batch normalization is applied to further optimize the findings and hasten the network’s convergence. “Fully linked layers” of 64 neurons are used. Softmax classifier is used at the output layer.

## 4. Results and Discussion

Because of the large dimensionality of deep neural networks, solving the challenge of choosing the parameters that should be used to train them in order to achieve the maximum possible performance is a challenging optimization problem. As a direct consequence of this, techniques based on stochastic optimization are utilized frequently. In this work, the adaptive moment estimation (ADAM) optimizer is used using the learning parameters that are laid out in Table 3. These parameters were determined by keeping an eye on the test results for validation, and they were used for all networks for easy comparison of their outputs and computational costs.

In order to assess the effectiveness of the suggested CAD framework, the Dice similarity coefficient (Dice) [97], accuracy, and sensitivity have been utilized. DSC is a performance indicator that can be used to evaluate how significant the autonomous segmentation actually is. The Dice coefficient, depicted by Equation (1), is a measurement that determines how similar two sets of data are to one another. The primary application for it is in the analysis of the segmentation models. We put it to use to determine how closely the segmented image and the ground truth image were related to one another.

(1) $$\mathrm{Dice}\;=\;\frac{2\mathrm{TP}}{2\mathrm{TP}\;+\;\mathrm{FP}\;+\;\mathrm{FN}}$$

*TP* is for “true positive,” *FP* stands for “false positive,” which indicates that the number was actually negative, but our model predicts it as positive, and *FN* stands for “false negative.”

Sensitivity is utilized for the purpose of predicting the real positive cases that were predicted as positive. It is also referred to as recall, and it is expressed as Equation (2).

(2) $$\mathrm{Sensitivity}\;=\;\frac{\mathrm{TP}}{\mathrm{TP}\;+\;\mathrm{FN}}$$

The accuracy refers to how well the predicted value and the actual value match up with one another. It is utilized in the method of performance analysis of the system. It is expressed as Equation (3), specifically.

(3) $$\mathrm{Accuracy}\;=\;\frac{\mathrm{TP}\;+\;\mathrm{TN}}{\mathrm{TP}\;+\;\mathrm{TN}\;+\;\mathrm{FP}\;+\;\mathrm{FN}}$$

Experiments were performed on three-dimensional MR images of a brain tumor. Images in three dimensions are those whose colors are produced by combining the red, green, and blue color channels. The information for each of the three channels—red, green, and blue—is stored in the image’s individual pixels. By providing the height, breadth, and depth dimensions, we are able to conceptualize a three-dimensional image. As stated in the dataset section, there are four types of tumor necrosis, edema, enhancing, and non-enhancing tumors, and the MRI sequences used to detect these tumors are FLAIR, T1, T1c, and T2. To detect these cancers, the image annotation is provided for all of these sequences. There is just one annotated image that includes all of these kinds as intratumor areas.

**Figure 2:**
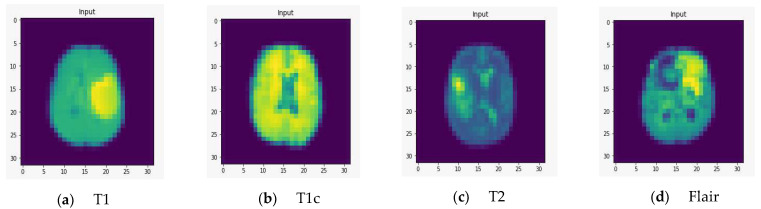
Rendering of T1, T1c, T2, and FLAIR at a resolution of 32 × 32 × 32.

**Figure 3:**
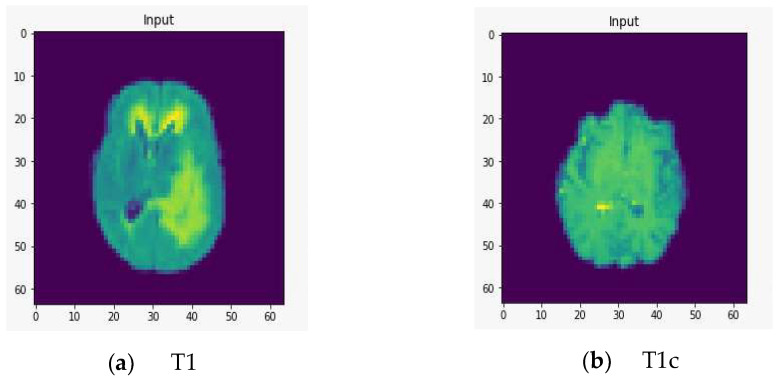
Rendering of T1, T1c, T2, and FLAIR at a resolution of 64 × 64 × 64.

**Figure 4:**
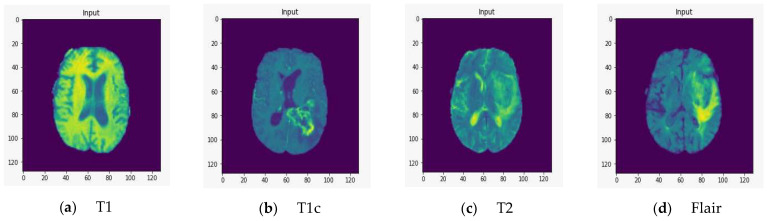
T1, T1c, T2, and FLAIR rendered at a resolution of 128 × 128 × 128.

To demonstrate the quality of input images, we represent several renderings of the input images in various sizes (Figure 2, Figure 3 and Figure 4). Figure 2 displays the visualization of HGG BRATS data for the MR sequences of T1, T1c, T2, and FLAIR at a resolution of 32 × 32 × 32. Images were transformed in the preprocessing phase. Tumor details were more easily discerned by viewing the identical pictures in a 64 × 64 × 64 matrix (Figure 3). A clearer view of brain tumor images with dimension of 128 × 128 × 128 is presented in Figure 4. The illustration depicts the afflicted area of brain that may be evaluated better than the prior dimensions.

**Figure 5:**
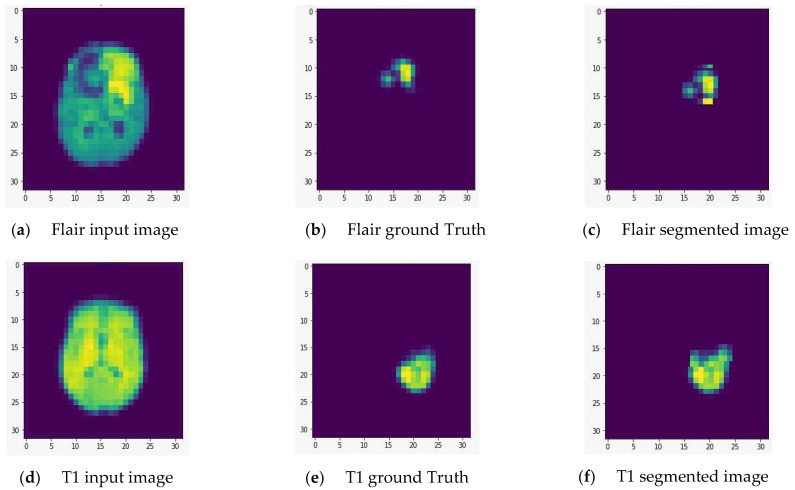

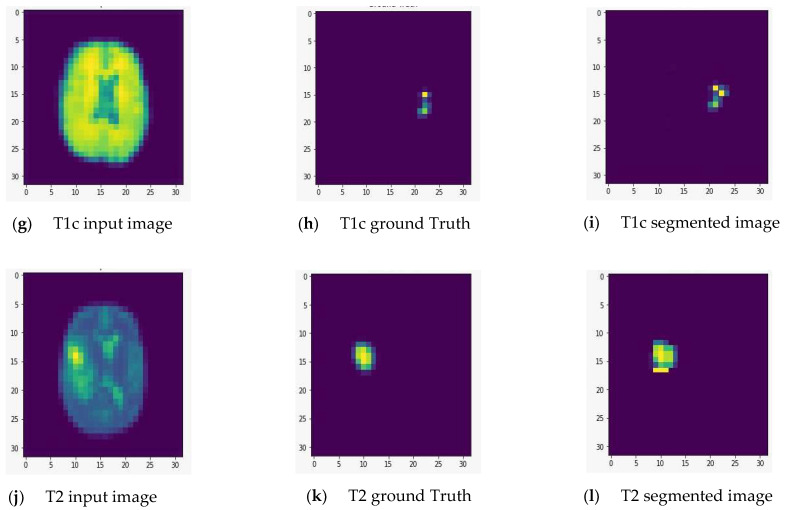
Segmentation of high-grade gliomas (HGG) from four MRI sequences (FLAIR, T1, T1C, and T2), respectively, using the U-Net model. Left column: input image; middle column: ground truth images; right column: segmented images.

**Figure 6:**
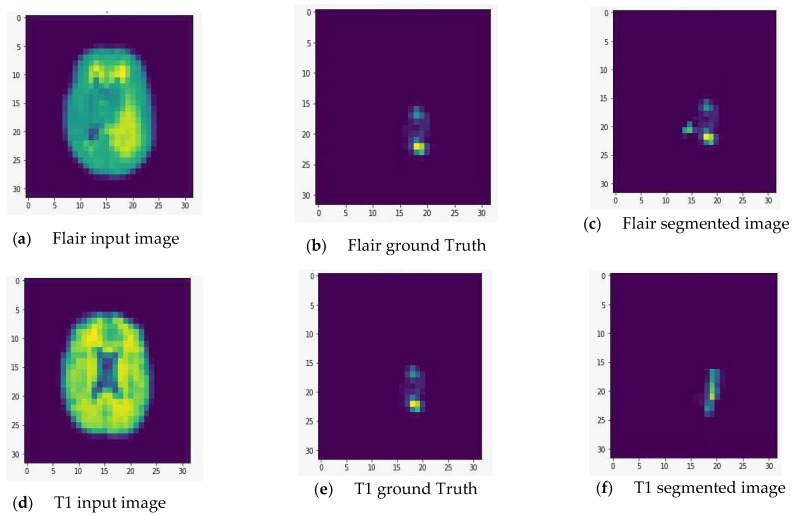

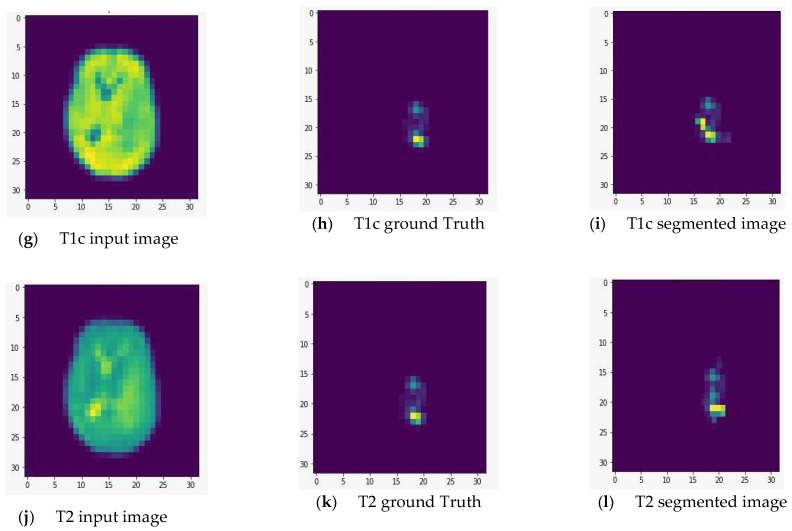
Segmentation of low-grade gliomas (LGG) from four MRI sequences (FLAIR, T1, T1C, and T2), respectively, using the U-Net model. Left column: input image; middle column: ground truth images; right column: segmented images.

We fed the preprocessed images into the U-Net model, which segmented out the brain tumor regions. At first look, the model appears to be divided into two phases: encoding and decoding. The goal of the encoding is to reduce spatial information while improving feature mapping by utilizing various blocks of the convolution and max-pooling layers. Following the encoding phase, the decoding step uses upsampling, convolution, and concatenation layers in blocks to split the input image into two parts—the tumor and the background. As a result, the suggested model will provide us with a segmented view of the brain tumor region. We conducted the experiment on both high-grade gliomas (HGG) and low-grade gliomas (LGG). Figure 5 and Figure 6 illustrate the segmentation of HGG and LGG brain tumors from four MRI sequences (FLAIR, T1, T1C, and T2), respectively, using the U-Net model. Each MRI sequence (FLAIR, T1, T1C, and T2) is displayed in three different views: the input view, the ground truth view, and the segmented view.

By calculating the Dice score and the sensitivity performance metrics, we were able to assess the feasibility of the U Net architecture for the task of segmenting brain tumors for HGG and LGG cases. The results that were retrieved were accomplished through the utilization of a 10-fold cross-validation test. Table 4 provides an illustration of the cross validation test by outlining the retrieved Dice score, sensitivity, and accuracy metrics for the complete type of the BT during the course of a 10-fold test.

The attained results for all types of BT are summarized in Table 5 for the segmentation and classification of several brain tumor regions, including complete, core, and enhancing, for both low-grade gliomas and high-grade gliomas. As can be seen in Table 5, the greatest possible values for the DSC, the sensitivity, and the classification accuracy for HGG patients in the complete brain tumor region are 88.8%, 89.4%, and 88.6%, respectively. These figures represent the best conceivable values in each of these categories. The learning model is adequate when seen through the lens of these values. The values 84.7%, 80%, and 83.1% are produced for the same measures when they are applied to the cases of low-grade gliomas that are found in the same location. The learning model’s accuracy in detecting malignancies in other brain tumor regions, such as the core and enhancing brain tumors, is lower than the values achieved for the complete brain tumor region. This is because the core and enhancing brain tumors are part of the complete brain tumor region. The obtained values for the DSC score, sensitivity, and classification accuracy for the core brain tumor are 83.2%, 80%, and 85.1% for high-grade gliomas, and 70.5%, 69%, and 71.5% for low-grade gliomas, respectively. In addition, the obtained values for the DSC score, sensitivity, and classification accuracy for the enhancing brain tumor are as follows: 71.8%, 73%, and 74.31% in the high-graded gliomas, and 60.2%, 64.3%, and 65.4% in the low-graded glioma.

We evaluated the relevance of the U-Net–CNN approach by comparing its accuracy to that of the most advanced segmentation and classification systems for brain tumor MRI scans by utilizing the BRATS 2015 database. This allowed us to determine how significant the U-Net–CNN approach is. The comparison between the suggested method and the existing standard of practice is presented in Table 6. The obtained values for the DSC and sensitivity performance metrics are significant compared to the current state of the art for deep learning-based segmentation of brain tumors in the public BRATS 2015 benchmarking dataset. This comparison takes place using the dataset that was made public in 2015.

When we assess the efficacy of our presented framework in light of the studies that were covered earlier in this part, we discover the following. The newly developed framework has achieved a level of performance that is superior to that accomplished by Vinay Rao et al. [69], Mengqiao et al. [73], and Casamitjana et al. [71]. When contrasted with the work done by Sergio Pereira et al. [70], Dong et al. [72], Heng Wang [74], Cui et al. [75], Lang et al. [76], Li et al. [77], and Peng et al. [78], the presented framework has acquired somewhat higher values for the Dice score, sensitivity, and accuracy.

## 5. Conclusions

This research introduces a unified framework for CAD of brain MRI images, with support from U-Net and deep convolutional neural networks. Image preprocessing and enhancement, brain skull stripping, brain tumor segmentation, feature extraction and selection, and benign/malignant tumor classification are the five basic stages of this type of computer-aided diagnosis system. We have created a model that is based on deep learning techniques so that automatic tumor segmentation and detection are performed using MRI scans of the brain. The model has two phases. In the first phase, ROIs are segmented. In the second phase, the tumor is classified into one of four categories: necrosis, edema, enhancing, or nonenhancing. The U-Net model is applied to the BRATS dataset to segment the image into the regions of interest.

The introduced lightweight U-Net model is composed of four convolutional layers. Two convolutional layers, each with a filter size of 3 × 3, are separated by a stride size of 1 within each block. The proposed CNN model’s input layer takes 32 × 32 ROIs. Convolutional layers follow the input layer. Five convolutional layers are used to extract information from ROIs. We picked max pooling because we only care about the most informative tumor tissue traits. After each convolutional layer, a max-pooling layer improves accuracy. We used three kernels and two strides in convolutional layers. Convolution layers use ReLU. Padding protects data at edges. Inputs and outputs have identical padding. After each convolution layer, batch normalization optimizes the findings and speeds network convergence. Connected layers of 64 neurons are used, as well as output layer Softmax classifier. In conclusion, the obtained results showed that the proposed model achieves better performance than existing models in terms of DSC and sensitivity on segmentation results and classification accuracy as well.

## 6. Limitations and Future Work

There is always opportunity for progress in the realm of research, and the medical sciences in particular have a pressing need for a highly precise model. The model can be applied to other datasets to extract and categorize the tumor types, expanding the suggested work of tumor identification in the medical area and assisting clinicians. There are a variety of ways in which this model could be improved. First, the U-Net model we used for tumor segmentation in BRATS 15 is transferable to the newer versions of the dataset that can be downloaded from the Multimodal BRATS website. BRATS 20, the latest and greatest edition, now features 3D graphics. The model can be adjusted to fit new datasets. Improved segmentation results on the BRATS 20 dataset are attainable by modifying the model’s featured layers. Second, different activation functions and CNN model layers can be tried out to find what produces the most reliable predictions. One can also offer the hybrid model by making use of the already existing one. Tumor detection in 3D images may potentially benefit from the U-Net model trained with GNN. Additional preprocessing techniques can be used for the hybrid model as a last resort for improved segmentation and classification results utilizing the current model.

## Figures and Tables

**Table 2:** The distribution of BT data throughout the several classes that are used in training, validation, and testing.

	High Grade Gliomas MRIs	Low Grade Gliomas MRIs
Training (70%)	616	120
Validation (15%)	132	28
Testing (15%)	132	28

**Table 3:** Hyperparameters for training the proposed model.

Hyperparameter	Value
Initial Learning rate	$10^{-3}$
No. of Epochs	200
Image Batch size	16
L2-Regularization	0.0004

**Table 4:** Evaluation of the proposed U-Net–CNN over 10-fold tests.

Fold	HGG			LGG		
	Dice Score (%)	Sensitivity (%)	Classification Accuracy (%)	Dice Score (%)	Sensitivity (%)	Classification Accuracy (%)
Fold 1	87.16%	88.61%	85.54%	80.5%	77.02%	80.04%
Fold 2	89.1%	88.92%	88.76%	85.3%	79.5%	79.96%
Fold 3	87.98%	89.8%	89.03%	83.6%	80.64%	80.2%
Fold 4	88.74%	89.53%	89.2%	82.1%	81.2%	81.4%
Fold 5	88.95%	88.76%	88.12%	85.84%	79.14	83.5%
Fold 6	88.97%	88.81%	89.98%	86.23%	80.65%	84.6%
Fold 7	88.47%	89.5%	88.73%	86.41%	80.12%	86.73%
Fold 8	88.62%	90.14%	88.91%	85.33%	80.79%	84.1%
Fold 9	89.68%	89.94%	87.66%	85.98%	80.2%	85.9%
Fold 10	89.83%	89.97%	90.22%	85.96%	80.74%	85.3%
Avg. (%)	88.8	89.4	88.6	84.7	80	83.1

**Table 5:** The attained Dice score, sensitivity, and accuracy metrics of the U-Net architecture for the task of segmenting and classifying the brain tumors for HGG and LGG cases in the BRATS 2015 MRI scans.

Brain Tumor Regions	HGG			LGG		
	Dice Score (%)	Sensitivity (%)	Classification Accuracy (%)	Dice Score (%)	Sensitivity (%)	Classification Accuracy (%)
**Complete**	88.8%	89.4%	88.6%	84.7%	80%	83.1%
**Core**	83.2%	80%	85.1%	70.5%	69%	71.5%
**Enhancing**	71.8%	73%	74.3%	60.2%	64.3%	65.4%

**Table 6:** Comparing the attained segmentation, and classification accurateness to the state-of-the-art brain tumor MRI scans using the BRATS 2015 database.

Author	Techniques	Task	BT Type	Accuracy	Dice Score	Sensitivity	Specificity
Vinay Rao et al. [69]	CNN	Classification	HGG	67%	-	-	-
Sergio Pereira et al. [70]	DCNN	Segmentation	HGG	-	87%	86%	-
Casamitjana et al. [71]	3D CNN	Segmentation	HGG	-	84%	-	-
Dong et al. [72]	U-Net	Segmentation	HGG	-	86%	-	-
Mengqiao et al. [73]	3D CNN	Segmentation	HGG	-	84%	82%	
Heng Wang [74]	CNN	Classification	HGG	86.0%	-	85.7%	86.5%
Cui et al. [75]	Cascade DCNN	Segmentation	HGG	-	89%	80%	-
Lang et al. [76]	CNN, SVM	Classification	HGG	88%	-	-	-
Li et al. [77]	Modified U-Net model	Segmentation	HGG	-	89%	-	-
Peng et al. [78]	3D U-Net model	Segmentation	HGG	-	85% (Whole BT) 72% (Core BT) 61% (Enhancing BT)	-	-
Proposed method	U-Net and CNN Cascaded framework	End-to-end system for the segmentation and classification of BT	HGG	88.6% (Whole BT) 85.1% (Core BT) 74.3% (Enhancing BT)	88.8% (Whole BT) 83.2% (Core BT) 71.8% (Enhancing BT)	89.4% (Whole BT) 80% (Core BT) 73% (Enhancing BT)	-
			LGG	83.1% (Whole BT) 75.5% (Core BT) 65.4% (Enhancing BT)	84.7% (Whole BT) 70.5% (Core BT) 60.2% (Enhancing BT)	80% (Whole BT) 69% (Core BT) 64.3% (Enhancing BT)	-

## Data Availability

Not applicable.
